# Impact of a Longer-Term Physical Activity Intervention on Inflammatory and Oxidative Stress Biomarkers in Older People with Metabolic Syndrome

**DOI:** 10.3390/antiox15020151

**Published:** 2026-01-23

**Authors:** Maria Magdalena Quetglas-Llabrés, Margalida Monserrat-Mesquida, Silvia García, Marina Ródenas-Munar, David Mateos, Lucía Ugarriza, Cristina Gómez, Antoni Sureda, Cristina Bouzas, Josep A. Tur

**Affiliations:** 1 Research Group on Community Nutrition & Oxidative Stress, Institute of Research on Health Sciences (IUNICS), University of the Balearic Islands, Guillem Colom Bldg, 07122 Palma de Mallorca, Spain; 2 CIBER Fisiopatología de la Obesidad y Nutrición (CIBEROBN), Instituto de Salud Carlos III (ISCIII), 28029 Madrid, Spain; 3 Health Research Institute of the Balearic Islands (IdISBa), 07120 Palma de Mallorca, Spain; 4 C.S. Camp Redó, IBSalut, 07010 Palma de Mallorca, Spain; 5 Clinical Analysis Service, University Hospital Son Espases, 07120 Palma de Mallorca, Spain

**Keywords:** metabolic syndrome, physical activity, oxidative stress, inflammation

## Abstract

Metabolic syndrome (MetS) is characterised by cardiometabolic risk factors and is closely associated with increased oxidative stress and chronic low-grade inflammation. MetS is largely driven by adverse lifestyle behaviours, particularly physical inactivity, and regular physical activity is recognised as a central strategy for its prevention and management. This study aimed to assess the long-term impact of a five-year follow-up period of physical activity on oxidative stress, inflammatory biomarkers, and cardiometabolic health in adults with MetS. Forty participants diagnosed with MetS (50% men, aged 55–75 years) were selected and stratified into two groups: those who increased their physical activity and those who reduced it during the intervention. Physical activity was assessed using metabolic equivalent task minutes per week (MET·min/week), and evaluations were performed at baseline, 3 years, and 5 years. Participants who increased physical activity showed a progressive reduction in reactive oxygen species (ROS) produced by peripheral blood mononuclear cells (PBMCs), together with a decrease in plasma malondialdehyde (MDA). Antioxidant enzyme activities, including catalase and superoxide dismutase, exhibited a favourable long-term profile, with recovery or maintenance of higher activity levels by the end of follow-up, reflecting enhanced endogenous antioxidant defence. Inflammatory status improved and was characterised by a reduction in myeloperoxidase (MPO) activity and a sustained increase in plasma interleukin-15 (IL-15). These participants also showed reductions in body weight, body mass index (BMI), waist circumference, fasting glucose, and glycosylated haemoglobin A1c (HbA1c), consistent with improved insulin sensitivity and metabolic control. Participants who reduced physical activity tended to show unfavourable trajectories in several biomarkers. Increasing physical activity over time is associated with substantial improvements in redox balance, inflammatory status, and cardiometabolic health in adults with MetS. These findings reinforce the central role of physical activity as a fundamental therapeutic component within lifestyle interventions aimed at mitigating metabolic dysfunction and preventing MetS progression.

## 1. Introduction

Metabolic syndrome (MetS) is a cluster of risk factors that includes abdominal obesity, high blood pressure, dyslipidaemia, and high fasting glucose [1]. MetS was linked to a fivefold increased risk of developing type 2 diabetes mellitus (T2DM), as well as a two-fold increased long-term risk of cardiovascular disease (CVD) and cardiovascular mortality, and was associated with a higher rate of cancer worldwide [2,3]. The aetiology of MetS, although largely unknown, is believed to lie in a complex interaction between genetic predisposition and metabolic and environmental factors [4]. The risk of developing MetS increases with age, obesity, a history of diabetes, and certain diseases. Factors such as abdominal fat and conditions like fatty liver and cardiovascular problems significantly increase this risk [5].

The complex pathophysiological state of MetS is influenced by an individual’s genetic and epigenetic makeup, reflecting both inherited susceptibility and long-term regulatory adaptations, together with the predominance of a sedentary lifestyle over physical activity and other factors such as food quality and composition, as well as gut microbiota composition [1]. A recent study showed a positive association between long-term sedentary behaviour and increased risk of MetS [6]. MetS is increasingly associated with impaired metabolic flexibility and mitochondrial dysfunction, which limit the capacity to efficiently adapt substrate utilisation to energetic demands [7]. Excess caloric intake and poor dietary quality, particularly diets rich in long-chain saturated fatty acids and sugars, promote mitochondrial overload, oxidative stress, and low-grade inflammation, thereby exacerbating insulin resistance and metabolic dysregulation [8]. In contrast, lifestyle interventions such as regular physical activity and dietary patterns including the Mediterranean diet enhance mitochondrial function, improve metabolic flexibility, and attenuate oxidative and inflammatory stress, playing a central role in the prevention and management of MetS [8]. In addition, skeletal muscle acts as an active endocrine organ, releasing myokines in response to contraction that exert systemic anti-inflammatory effects. This exercise-induced muscle-derived signalling can counterbalance the pro-inflammatory phenotype of expanded adipose tissue, thereby modulating chronic low-grade inflammation characteristic of MetS [9].

Oxidative stress, an imbalance between reactive oxygen/nitrogen species and antioxidant defences, further amplifies metabolic dysfunction. While physiological levels of reactive species contribute to host defence and cell signalling, sustained excess perturbs redox homeostasis. Endogenous enzymatic defences include catalase (CAT) and superoxide dismutase (SOD). Lipid peroxidation yields malondialdehyde (MDA), widely used as an index of oxidative damage [10]. MetS is characterised by an increased oxidative stress situation and chronic low-grade inflammation [11]. Acute inflammatory responses are generally self-limited, but failure of resolution or persistent stimuli leads to chronic inflammation, implicated in obesity and MetS pathogenesis [12]. In obesity, expansion of white adipose tissue increases oxidative stress and promotes pro-inflammatory cytokine production, while anti-inflammatory adipokines decline [11]. In MetS, myeloperoxidase (MPO), a haem peroxidase predominantly expressed in neutrophils, generates hypochlorous acid, and serves both antimicrobial and pro-inflammatory roles; higher plasma MPO activity and falls with weight reduction [11].

Lifestyle factors, particularly physical activity, are key modifiable determinants of MetS risk. Sedentary behaviour is associated with a higher incidence of CVD and MetS [13], whereas structured lifestyle programmes improve insulin sensitivity, blood pressure, lipid profiles, and central adiposity, thereby reducing cardiometabolic risk [14]. Interleukin-15 (IL-15), although pro-inflammatory in some immunological contexts [15], is expressed in skeletal muscle and has been linked to enhanced fatty acid oxidation, reduced fat mass, improved insulin sensitivity, and attenuation of NF-κB signalling [16].

Although the beneficial effects of physical activity on MetS have been widely reported, long-term human data integrating oxidative stress, inflammatory biomarkers, and clinical outcomes across multiple time points remain scarce. Therefore, this study aimed to characterise the 3- and 5-year trajectories of redox-inflammatory biomarkers in adults with MetS, stratified according to changes in physical activity.

## 2. Methods

### 2.1. Study Design and Participants

A longitudinal study was conducted in 40 adults (55–75-year-old men and 60–75-year-old women) with MetS participating in a lifestyle intervention trial. MetS was defined according to the criteria of the International Diabetes Federation, the American Heart Association, and the National Heart, Lung, and Blood Institute. All participants were of European Caucasian ancestry, and diagnostic thresholds validated for this population were applied. Participants met at least three of the following criteria: (1) abdominal obesity (waist circumference ≥102 cm in men and ≥88 cm in women), (2) high triglyceride blood level (≥150 mg/dL) or current treatment for hypertriglyceridemia, (3) reduced high-density lipoprotein cholesterol (<40 mg/dL in men and <50 mg/dL in women), (4) high blood pressure (≥130/85 mmHg or antihypertensive treatment), and (5) high fasting plasma glycaemia (≥100 mg/dL) or current treatment for type 2 diabetes. Additional inclusion criteria included overweight or obesity (body mass index (BMI) ≥ 27 and <40 kg/m^2^) and the absence of clinically documented cardiovascular disease.

**Figure 1:**
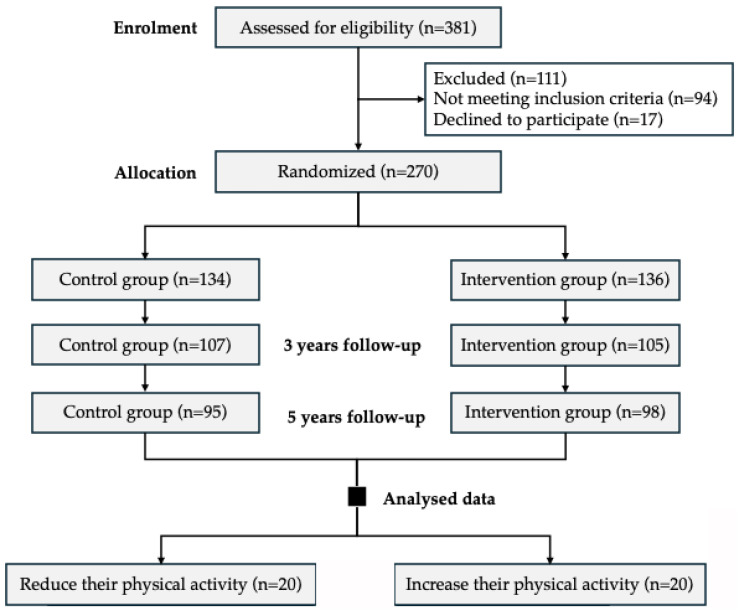
Flowchart of the study.

From an initial cohort of 381 participants screened for eligibility, 111 were excluded because they did not meet the inclusion criteria or declined to participate. A total of 270 participants were subsequently enrolled and randomly allocated in a 1:1 ratio to one of two intervention programmes: an intensive lifestyle intervention based on a low-calorie Mediterranean diet (MedDiet), physical activity promotion and behavioural counselling for weight loss, or a standard programme involving an energy-unrestricted MedDiet and conventional cardiovascular prevention advice (Figure 1).

For the current longitudinal analysis, a subsample of 40 participants was selected from the original cohort based on changes in physical activity during the intervention period. Individuals were reclassified according to their physical activity evolution, resulting in two groups: those who reduced (n = 20) and those who increased (n = 20) their physical activity level. Each group included an equal number of men and women (n = 10 per sex). The distribution of participants across the two intervention arms of the parent trial was balanced. All participants were evaluated at baseline, 3 years, and 5 years of follow-up for metabolic, oxidative stress, and inflammatory parameters, allowing the longitudinal assessment of lifestyle-induced changes in redox and inflammatory biomarkers in adults with MetS.

All participants were fully informed about the objectives and potential implications of the study and provided written informed consent. The study complied with the ethical principles of the Declaration of Helsinki and was approved by the Ethics Committee of Research of the Balearic Islands (reference CEIC-IB/2251/14PI).

### 2.2. Physical Activity Assessment

Physical activity was assessed using metabolic equivalents of task (METs) scores to estimate energy expenditure, according to established compendia of physical activity [17]. Participants reported the average duration and frequency of different activities, expressed in minutes per week, from which total MET·min/day were calculated.

### 2.3. Anthropometrics and Clinical Assessments

Certified dietitians assessed body weight and height using calibrated digital scales and a fixed wall stadiometer, respectively. Waist circumference, which indicates abdominal obesity, was determined midway between the lowest rib and the iliac crest with a flexible, non-elastic anthropometric tape. All anthropometric variables were recorded in duplicate to ensure accuracy. Body mass index (BMI) was obtained by dividing body weight (kg) by the square of height (m^2^), and waist-to-height ratio (WHtR) was calculated by dividing waist circumference (cm) by height (cm).

Systolic and diastolic blood pressure were measured three times using a validated semi-automatic oscillometric device (Omron HEM-705CP, Lake Forest, IL, USA) after a 5 min seated rest, allowing short intervals between measurements.

### 2.4. Blood Collection and Analysis

Venous blood samples were collected from all participants after a 12 h overnight fast. Blood was drawn from the antecubital vein into sterile vacutainer tubes both with and without ethylenediaminetetraacetic acid (EDTA) as an anticoagulant, to obtain plasma and serum, respectively. Serum samples were used for routine biochemical analyses, including glycaemia, HbA1c, triglycerides, HDL-cholesterol, LDL-cholesterol, and total cholesterol, which were performed using standard enzymatic methods at the Clinical Laboratory of Son Espases University Hospital (Palma, Spain).

Plasma and peripheral blood mononuclear cells (PBMCs) were isolated from fresh whole blood immediately after collection in EDTA-containing tubes. Plasma samples were obtained by centrifuging fresh whole blood at 1700× *g* for 15 min at 4 °C. PBMCs were purified using Ficoll-Paque PLUS reagent (GE Healthcare Bio-Sciences AB, Uppsala, Sweden). Briefly, 6 mL of whole blood were gently layered over 4 mL of Ficoll solution (ratio 1.5:1) and centrifuged at 900× *g* for 30 min at 4 °C. Following centrifugation, both the plasma and Ficoll layers were removed, and the PBMC layer was carefully collected. The PBMC fraction was washed with phosphate-buffered saline (PBS; pH 7.4) and centrifuged again at 900× *g* for 10 min at 4 °C. Aliquots of plasma and isolated PBMCs were immediately stored at −80 °C until further biochemical and molecular analyses.

### 2.5. Dietary Assessment

Dietary intake was assessed by trained dietitians using a validated 143-item semi-quantitative food frequency questionnaire (FFQ) developed for the Spanish population [18]. The FFQ evaluated habitual food consumption during the previous year, and energy and nutrient intakes were calculated using Spanish food composition tables and a validated computer programme [19].

Adherence to the MedDiet was determined through a 17-item questionnaire [20], in which each item reflecting a typical component of the Mediterranean dietary pattern was scored as 1 (compliance) or 0 (non-compliance). The total score ranged from 0 (minimum adherence) to 17 (maximum adherence).

This information was used to describe participants’ dietary patterns and to verify that both physical activity groups exhibited comparable adherence to the MedDiet and energy intake throughout follow-up.

### 2.6. Oxidative Stress and Inflammatory Parameters

Plasma enzymatic activities of catalase (CAT), superoxide dismutase (SOD), and myeloperoxidase (MPO) were quantified at 37 °C using a Shimadzu UV-2011 spectrophotometer (Shimadzu Corporation, Kyoto, Japan). CAT activity was determined by monitoring the rate of hydrogen peroxide (H_2_O_2_) decomposition at 240 nm, following Aebi’s spectrophotometric method [21]. SOD activity was assessed by measuring the inhibition of cytochrome C reduction at 550 nm according to the modified McCord and Fridovich protocol [22]. MPO activity was evaluated through the oxidation of guaiacol, with the formation of tetraguaiacol measured at 470 nm [23].

Malondialdehyde (MDA) was determined using a specific colorimetric assay kit (Sigma-Aldrich Merck^®^, St. Louis, MO, USA) according to the manufacturer’s instructions. In brief, plasma samples or standards were incubated with *N*-methyl-2-phenylindole dissolved in a methanol/acetonitrile (1:3) solution. Subsequently, 75 µL of 12 N hydrochloric acid (HCl) was added, and the mixture was incubated for 60 min at 45 °C. Absorbance was read at 586 nm using a microplate reader (Epoch, BioTek^®^ Instruments GmbH, Bad Friedrichshall, Germany). MDA levels were calculated from a standard calibration curve prepared with known concentrations.

Plasma concentrations of interleukin-15 (IL-15) were measured using commercial ELISA kits (EH0177, Wuhan Fine Biotech Co., Wuhan, China) according to the manufacturer’s guidelines. The intra-assay and inter-assay coefficients of variation (CVs) were <10% and <12%, respectively. Absorbance was measured at 450 nm using a microplate reader (Epoch, BioTek^®^ Instruments GmbH, Bad Friedrichshall, Germany).

Reactive oxygen species (ROS) generation in PBMCs was quantified after stimulation with lipopolysaccharide (LPS, 100 µg/mL in phosphate-buffered saline) from *Escherichia coli* (Sigma-Aldrich, St. Louis, MO, USA). Cell suspensions containing approximately 6 × 10^5^ cells per 50 µL were placed in 96-well plates, followed by the addition of 50 µL LPS to the respective wells. The fluorogenic probe 2′,7′-dichlorofluorescein diacetate (DCFH-DA, 61.6 µM in Hanks’ Balanced Salt Solution) was then added. Fluorescence (excitation 480 nm; emission 530 nm) was recorded continuously for 60 min at 37 °C using a FLx800 Microplate Fluorescence Reader (BioTek Instruments, Winooski, VT, USA). ROS production was calculated from a standard curve prepared under identical experimental conditions.

### 2.7. Statistics

Statistical analyses were performed using the Statistical Package for the Social Sciences (SPSS, version 29.0; IBM Corp., Chicago, IL, USA). Data are expressed as mean ± standard deviation (SD), and statistical significance was set at *p* < 0.05. The Shapiro–Wilk test was applied to verify the normal distribution of the variables. A general linear model (GLM) for repeated measures was used to assess differences across the three time points (baseline, 3 years, and 5 years) and between physical activity groups, adjusting for age and sex. When significant effects were detected, pairwise comparisons were performed using the Bonferroni correction to control for multiple testing.

## 3. Results

The mean age was 63.4 ± 5.9 years in the group that reduced physical activity and 63.9 ± 5.0 years in those that increased it (*p* = 0.982). Medication use and comorbidities were similar between groups. The prevalence of hypertension was 5% in both groups, while type 2 diabetes was present in 40% and 45% of participants in the reduced and increased activity groups, respectively (*p* = 1.000). The proportion of participants using lipid-lowering, antihypertensive, or glucose-lowering medications did not differ significantly between groups (all *p* > 0.05).

**Figure 2:**
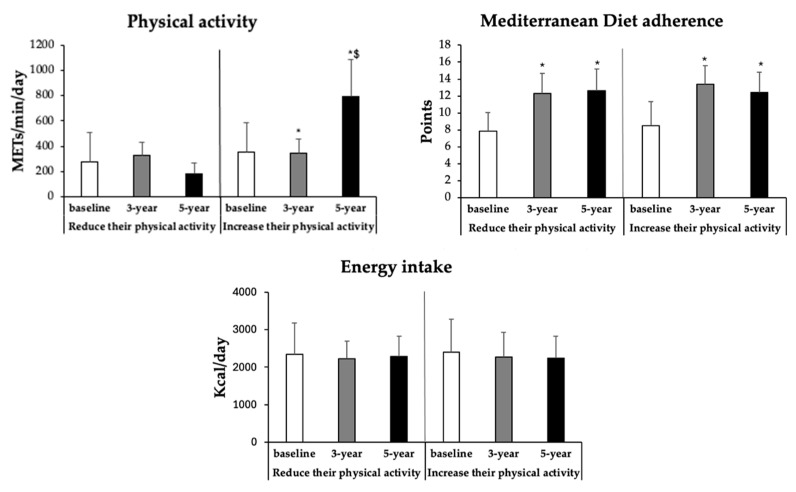
Temporal changes in physical activity, Mediterranean diet adherence, and energy intake in participants who reduced or increased their physical activity at baseline, 3 years, and 5 years. Results are expressed as mean ± SD. Two-way analysis of covariance (ANCOVA) was performed after adjustments for age and sex. Data points are significant, *p* < 0.05. * Difference in means between participants in time with respect to baseline. ^$^ Difference in means between groups (reduce or increase their physical activity) at the same time.

As shown in Figure 2, physical activity levels (MET·min/day) significantly increased across time in the group that enhanced physical activity, while they decreased in those that reduced it (*p* < 0.001). Adherence to the MedDiet improved significantly from baseline to 3 and 5 years in both groups (*p* < 0.05), with no significant differences between them. Energy intake decreased slightly during the intervention, with no significant differences observed either over time or between groups (*p* = 0.820).

Baseline anthropometric and biochemical characteristics are summarised in Table 1. At the start of the study, no significant differences were observed between groups. After 3 years, both groups showed a reduction in body weight compared with baseline. By the fifth year, participants who increased physical activity maintained a lower body weight (81.4 ± 11.0 kg) than those who reduced physical activity (88.0 ± 17.2 kg; *p* < 0.001). BMI followed a similar pattern; the increased physical activity group exhibited a significant decrease at 5 years (31.5 ± 3.9 kg/m^2^) compared with baseline, while BMI increased slightly in the reduced activity group. Waist circumference and the waist-to-height ratio remained stable throughout the study in both groups (*p* > 0.05). Systolic and diastolic blood pressure did not differ significantly between groups at any time point (*p* > 0.05).

Regarding glucose metabolism, fasting glucose levels were significantly lower at 5 years in the increased physical activity group (99.8 ± 18.7 mg/dL) compared with those with reduced physical activity (121.7 ± 27.5 mg/dL; *p* < 0.001). HbA1c levels increased over time in the reduced physical activity group but decreased significantly in those with increased physical activity at 5 years. Lipid parameters (triglycerides, HDL-cholesterol, and LDL-cholesterol) did not differ significantly between groups over the follow-up period. However, within-group analyses revealed a significant reduction in LDL-cholesterol between years 3 and 5 in participants who increased their physical activity. The distribution of macronutrient intake (% energy from carbohydrates, proteins, and lipids) remained relatively constant throughout the intervention in both groups, with no significant between-group differences (all *p* > 0.05).

This study provides long-term longitudinal evidence on within-individual trajectories of redox and inflammatory biomarkers over five years in adults with MetS, according to changes in habitual physical activity. Oxidative stress biomarkers are presented in Table 2. Reactive oxygen species (ROS) production in PBMCs decreased significantly over time in both groups (*p* < 0.001), with the lowest values observed after 5 years. In plasma, CAT, SOD, and MPO enzyme activities all showed significant changes across time. CAT activity significantly decreased at 3 years compared with baseline in both groups and remained stable thereafter. SOD activity increased significantly from baseline to 3 and 5 years in both groups, with higher levels at 5 years in the increased activity group. MDA levels decreased significantly over time in both groups (*p* = 0.001), reaching lower values at 5 years in the increased activity group (0.450 ± 0.128 nM) compared with the reduced activity group (0.573 ± 0.150 nM).

**Figure 3:**
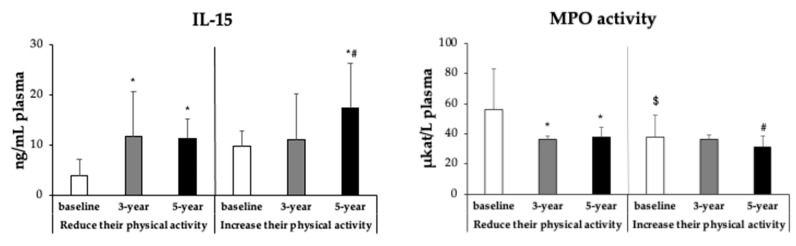
Plasma concentrations of interleukin-15 (IL-15) and myeloperoxidase (MPO), considered inflammatory biomarkers, are presented for participants with MetS at baseline, and after 3 and 5 years of intervention, according to changes in physical activity. Results are expressed as mean ± SD. Two-way analysis of covariance (ANCOVA) was performed after adjustments for age and sex. Data points are significant, *p* < 0.05. * Difference in means between participants in time with respect to baseline. ^#^ Difference in means between participants in time with respect to 3 years. ^$^ Difference in means between groups (reduce or increase their physical activity) at the same time.

Inflammatory biomarker data are shown in Figure 3. IL-15 plasma concentrations increased significantly from baseline to 3 years in both groups (*p* < 0.05). At 5 years, IL-15 levels remained high in both groups, with significantly higher values in participants who increased physical activity (17.4 ± 8.80 ng/mL) compared with those who reduced it (11.3 ± 3.86 ng/mL; *p* < 0.001). Similarly, plasma MPO activity decreased progressively from baseline to 3 and 5 years, with significantly lower values at 5 years in the increased physical activity group compared with the reduced physical activity subjects.

## 4. Discussion

Unlike most previous studies based on cross-sectional comparisons or short-term interventions, the present analysis captures divergent within-individual trajectories over five years, allowing the temporal association between changes in physical activity and redox–inflammatory adaptations to be examined. The findings show that participants who increased their physical activity throughout the follow-up exhibited a more favourable oxidative and inflammatory profile, together with improved metabolic outcomes, compared with those who reduced their activity levels. Physical activity, therefore, emerges as the principal discriminating factor between groups, as adherence to the Mediterranean diet and total energy intake were comparable at baseline and evolved similarly over the intervention period. Baseline physical activity levels in both groups were already within current public health recommendations. However, large dose–response meta-analyses indicate that the greatest reductions in cardiometabolic risk occur at substantially higher volumes of physical activity [24]. In this context, the pronounced divergence observed at five years may partly explain the greater metabolic and oxidative improvements detected in the physically active group.

The longitudinal evolution of oxidative stress biomarkers revealed a sustained improvement in redox balance over the five-year period, particularly among participants who increased their physical activity. ROS production in PBMCs showed a marked and progressive decline. This pattern aligns with evidence indicating that physical activity reduces NADPH oxidase activity and other upstream sources of oxidative stress [25], while enhancing mitochondrial respiration, ATP production, and overall mitochondrial efficiency [25,26]. Such adaptations contrast sharply with the generation of superoxide and mitochondrial dysfunction typically observed in sedentary individuals with MetS [27]. Although acute exercise may transiently raise ROS, long-term engagement in regular activity induces beneficial redox adaptations and strengthens endogenous antioxidant defences [28].

Catalase activity followed a biphasic trajectory, characterised by an initial decline at the three-year assessment in both groups, followed by a recovery at five years that was most pronounced among participants who increased their physical activity. This early decrease may reflect an adaptive downregulation in response to the rapidly shifting redox environment observed during the first stages of the intervention, when the marked reduction in ROS production likely reduced the oxidative stimulus required to sustain catalase activation. Under conditions of diminished ROS availability, catalase activity may transiently fall due to substrate limitation or feedback-mediated adjustments while the antioxidant system recalibrates to a lower oxidative load. As redox homeostasis becomes progressively stabilised, catalase activity rises again, consistent with the delayed activation of Nrf2- and PGC-1α-dependent transcriptional pathways that are engaged once sustained, low-grade redox signalling is re-established [29,30]. Although these signalling pathways were not directly assessed in the present study, the observed longitudinal biomarker patterns are consistent with exercise-induced adaptations previously described in human and experimental studies. The more complete enzymatic recovery observed in the active group suggests that regular physical activity provides sufficient mitochondrial and cytosolic stimulation to preserve long-term antioxidant adaptability and to maintain a dynamic redox-responsive phenotype.

Superoxide dismutase activity exhibited a different pattern, with a marked increase from baseline to three years that was maintained at the five-year follow-up, particularly among participants who increased their physical activity. This sustained elevation aligns with the role of SOD as the primary mitochondrial and cytosolic defence against superoxide anion. Regular physical activity is known to induce repeated, moderate bursts of ROS that act as molecular signals to enhance antioxidant gene expression, thereby strengthening endogenous defence mechanisms. Such adaptations may be mediated by canonical stress-responsive pathways, including Nrf2, NF-κB, and PGC-1α, which collectively promote improved mitochondrial function and systemic redox equilibrium [31,32]. These findings mirror observations in trained older adults, who exhibit higher SOD and CAT activities compared with sedentary individuals [31].

Interestingly, despite reducing their physical activity over time, the less active group did not exhibit a decline in CAT or SOD activities. This relative stability may reflect the compensatory influence of the MedDiet, rich in polyphenols, vitamins, and other bioactive compounds with recognised antioxidant properties. Evidence suggests that adherence to this dietary pattern can attenuate oxidative stress in individuals with obesity and metabolic disorders, thereby partially offsetting the loss of exercise-induced antioxidant stimulation [33]. Nonetheless, the divergence observed at the five-year follow-up, particularly the greater enzymatic recovery in the physically active group, highlights the unique and irreplaceable role of sustained physical activity in shaping long-term antioxidant resilience.

Moreover, plasma levels of MDA, a marker of lipid peroxidation, significantly decreased in the group that increased their physical activity, while remaining unchanged in those who reduced it. Elevated MDA concentrations are characteristic of individuals with MetS [11,34], and their reduction reflects improved oxidative balance and lipid membrane stability. In line with these observations, long-term lifestyle interventions that include sustained increases in physical activity have been shown to result in reductions in MDA, as evidenced in a 6-year nutritional and lifestyle programme in adults with MetS in which decreases in MDA accompanied improvements in BMI and metabolic status [35]. By contrast, shorter interventions have yielded more heterogeneous results. A 20-week lifestyle modification programme combining nutritional counselling and regular physical activity in 60 MetS subjects did not produce significant changes in MDA, although concentrations were positively associated with fasting glucose and HOMA-IR [36]. Likewise, a 6-month aerobic exercise intervention in sedentary older adults resulted in decreased AOPP but persistently higher MDA levels in participants with MetS [37]. Taken together, these findings suggest that meaningful reductions in lipid peroxidation in MetS may require sustained, long-term improvements in physical activity, supporting the notion that prolonged metabolic adaptation is necessary to restore redox balance.

Regarding inflammatory status, a marked reduction in plasma myeloperoxidase (MPO) activity was observed in participants who increased their physical activity, whereas the less active group showed an upward trend. MPO, a haem-containing peroxidase released from activated neutrophils, catalyses the formation of hypochlorous acid and exerts potent pro-inflammatory and pro-oxidative effects [38]. Elevated MPO activity has been correlated with adiposity, waist circumference, insulin resistance, and other metabolic disturbances typical of MetS [39]. Lifestyle interventions combining dietary modification and physical activity have shown reductions in MPO-derived oxidation products after 12 weeks in individuals with MetS [40]. Given the functional heterogeneity of neutrophil populations, circulating MPO activity likely reflects the balance between pro-inflammatory and regulatory neutrophil phenotypes rather than uniform changes in neutrophil function. The reduction observed in the physically active group in the present study therefore suggests decreased neutrophil activation and improved vascular redox-inflammatory status, both of which are associated with lower cardiometabolic risk.

In parallel, plasma levels of IL-15 increased significantly in the physically active group across the follow-up period. IL-15 is a myokine produced by skeletal muscle during contraction that plays a dual metabolic and immunomodulatory role. In this context, active skeletal muscle adopts an anti-inflammatory endocrine phenotype that counteracts the pro-inflammatory milieu typically associated with expanded adipose tissue in MetS, thereby contributing to the restoration of systemic inflammatory balance [9]. It enhances glucose uptake by skeletal muscle through GLUT4 translocation via the JAK3/STAT3 pathway, increases fatty acid oxidation, and reduces adipose tissue mass by redirecting energy substrates towards muscle metabolism [41,42]. Although evidence regarding changes in circulating IL-15 with regular physical activity remains inconsistent, with some studies reporting reductions in basal IL-15 concentrations following habitual exercise [43] and others showing no clear between-group differences despite associations with changes in body composition [44], acute bouts of exercise are known to elicit transient elevations in circulating IL-15 and related cytokines [45,46]. Within this context, the sustained increase observed in the physically active group of the present study may reflect long-term adaptations in muscle-derived myokine secretion associated with improved fitness and reduced adiposity. Overall, these findings support the role of IL-15 as a key mediator of the metabolic benefits induced by regular physical activity in individuals with MetS.

The improvement in oxidative and inflammatory profiles was accompanied by favourable changes in clinical and metabolic variables. Participants who increased their physical activity demonstrated reductions in BMI, waist circumference, fasting glucose, and HbA1c levels, consistent with enhanced insulin sensitivity and overall metabolic control. These effects are consistent with the literature showing that aerobic and resistance exercise programmes improve glucose homeostasis through upregulation of the GLUT4 transporter in skeletal muscle, increased mitochondrial efficiency, and reduced hepatic fat accumulation [47,48,49,50]. In line with our findings, previous research shows that regular physical activity does not consistently induce large reductions in total body weight, yet it remains essential for weight-loss maintenance and disproportionately reduces abdominal adiposity. This pattern is compatible with the modest but clinically relevant improvements observed in waist-related indices in the physically active group of our cohort [51,52,53].

The observed reductions in fasting glucose and HbA1c in the physically active group align with studies demonstrating that aerobic and concurrent training significantly decrease insulin concentrations, HOMA-IR, and glycaemic markers in overweight, obese, and metabolically impaired individuals [54,55]. Conversely, the absence of pronounced differences in lipid parameters between groups may be explained by the modest magnitude of exercise-induced changes in triglycerides, LDL-C, and HDL-C, which often require larger sample sizes to achieve statistical significance [54,56]. MetS can also be conceptualised as a continuous condition using composite severity scores that integrate multiple cardiometabolic components. However, in the present study, individual clinical variables were analysed separately to preserve physiological interpretability and to allow a clearer integration with the observed oxidative and inflammatory adaptations.

Interestingly, participants who reduced their physical activity did not exhibit a clear deterioration of MetS components over time. This stabilisation may be partially attributed to their sustained adherence to the hypocaloric MedDiet, which independently supports improvements in glycaemic control, lipid profile, and inflammatory status [38,57]. Nevertheless, the marked contrasts between groups underscore the central role of physical activity as the primary determinant of long-term metabolic improvement and redox–inflammatory regulation. While dietary adherence appeared comparable between groups throughout the intervention, only participants who increased their habitual physical activity displayed coordinated improvements across oxidative stress markers, inflammatory biomarkers, and key clinical parameters. From a translational perspective, these findings provide long-term human evidence linking sustained changes in habitual physical activity to coordinated oxidative, inflammatory, and metabolic adaptations, beyond the effects of diet alone. MedDiet components, such as polyphenols, have been shown to induce mild hormetic stimuli that enhance mitochondrial function, improve metabolic flexibility, and reinforce endogenous antioxidant and anti-inflammatory defence systems [58]. By capturing within-individual trajectories over five years, the present study extends previous short-term observations and generates testable hypotheses for future research addressing the intracellular signalling mechanisms underlying exercise-induced benefits in metabolic syndrome.

## 5. Strengths and Limitations of the Study

The current study has notable strengths. Foremost, it provides longitudinal evidence linking changes in physical activity to concurrent modifications in oxidative stress, inflammatory biomarkers, and clinically relevant metabolic parameters in adults with MetS. The repeated assessments at baseline, 3 years, and 5 years offer an unusually robust temporal perspective, allowing the characterisation of progressive biological adaptations to lifestyle modification rather than short-term or transient effects. The inclusion of an equal number of men and women within each physical activity group further reinforces internal comparability and reduces sex-related confounding. Moreover, the comprehensive profiling of redox and inflammatory markers, combined with detailed anthropometric and biochemical measurements, offers an integrated view of the physiological pathways through which physical activity may influence the progression of MetS.

Despite these strengths, certain limitations should be acknowledged. The relatively small sample size limits the statistical power for detecting more subtle associations, particularly in lipid parameters, and precludes additional stratified analyses. As the subsample was selected according to changes in physical activity rather than randomisation, the possibility of unmeasured behavioural or motivational factors influencing group allocation cannot be entirely dismissed. A further limitation is the absence of direct measurements of intracellular signalling pathways involved in redox and inflammatory regulation. Consequently, mechanistic interpretations are based on biomarker patterns and supported by existing literature rather than direct molecular evidence. Nevertheless, despite these constraints, the study yielded consistent and biologically plausible findings, reinforcing the central contribution of regular physical activity to the long-term metabolic and redox–inflammatory profile of individuals with MetS.

## 6. Conclusions

This five-year longitudinal analysis shows that sustained increases in physical activity exert a decisive impact on oxidative stress, inflammatory status, and metabolic health in individuals with MetS. Participants who enhanced their physical activity showed substantial reductions in ROS production, improved antioxidant enzyme activities, lower lipid peroxidation, and decreased neutrophil-derived inflammatory activity, together with increases in IL-15, a key myokine associated with muscle–metabolic crosstalk. These biochemical adaptations were accompanied by favourable changes in anthropometric and glycaemic parameters, underscoring the systemic benefits of regular physical activity. In contrast, individuals who reduced their physical activity exhibited a markedly attenuated response, despite maintaining MedDiet adherence. These findings highlight the central role of physical activity as the principal behavioural determinant of long-term redox–inflammatory balance and metabolic control within lifestyle interventions. Promoting sustained physical activity should therefore be a priority in clinical and public health strategies aimed at preventing the progression of MetS and improving cardiometabolic outcomes.

## Figures and Tables

**Table 1:** Characteristics of participants with MetS at baseline, as well as after 3-year and 5-year intervention.

		Reduce Their Physical Activity (n = 20)	Increase Their Physical Activity (n = 20)	*p*-Value
General characteristics				
	baseline	90.1 ± 17.0	83.8 ± 12.4	
Weight, kg	3-year	86.1 ± 17.5 *	85.7 ± 12.3	
	5-year	88.0 ± 17.2 *^,$^	81.4 ± 11.0 *^,$^	<0.001
	baseline	32.9 ± 3.5	32.2 ± 4.0	
BMI, kg/m^2^	3-year	32.6 ± 3.6	33.1 ± 4.5 *	
	5-year	33.4 ± 3.5 ^$^	31.5 ± 3.9 *^,$^	<0.001
	baseline	111.7 ± 8.9	107.1 ± 9.69	
Abdominal obesity, cm	3-year	109.5 ± 9.8	108.5 ± 10.6	
	5-year	109.2 ± 8.6	106.2 ± 8.60	0.074
	baseline	0.678 ± 0.05	0.664 ± 0.059	
WHtR	3-year	0.664 ± 0.05	0.673 ± 0.067	
	5-year	0.663 ± 0.05	0.659 ± 0.056	0.082
Systolic pressure, mmHg	baseline	141.9 ± 20.0	138.5 ± 19.9	
	3-year	133.2 ± 14.9	138.9 ± 17.0	
	5-year	142.0 ± 14.7	150.5 ± 18.6	0.213
Diastolic pressure, mmHg	baseline	79.5 ± 9.7	81.1 ± 7.4	
	3-year	76.1 ± 11.6	79.9 ± 8.0	
	5-year	79.2 ± 10.3	82.6 ± 8.8	0.733
Blood parameters				
	baseline	122.4 ± 41.5	114.3 ± 19.3	
Glucose, mg/dL	3-year	112.1 ± 27.8	112.8 ± 20.6	
	5-year	121.7 ± 27.5	99.8 ± 18.7 ^#,$^	<0.001
	baseline	6.27 ± 1.15	6.10 ± 0.55	
HbA1c, %	3-year	6.07 ± 0.72	6.04 ± 0.80	
	5-year	6.47 ± 0.73 ^#^	5.76 ± 0.66 ^$^	0.002
	baseline	149.5 ± 66.6	121.1 ± 38.2	
Triglycerides, mg/dL	3-year	128.4 ± 56.7	130.5 ± 43.8	
	5-year	138.0 ± 45.6	122.3 ± 33.5	0.077
HDL-cholesterol, mg/dL	baseline	45.7 ± 13.4	45.5 ± 8.3	
	3-year	47.1 ± 8.6	47.3 ± 8.6	
	5-year	46.7 ± 9.6	48.5 ± 11.0	0.709
LDL-cholesterol, mg/dL	baseline	111.6 ± 42.2	111.2 ± 32.8	
	3-year	113.2 ± 44.2	116.6 ± 30.7	
	5-year	111.5 ± 36.3	102.8 ± 30.7 ^#^	0.161
Dietary assessment				
	baseline	40.0 ± 7.9	38.3 ± 8.0	
Carbohydrates, %	3-year	40.8 ± 6.7	38.9 ± 5.7	
	5-year	37.7 ± 7.7	37.8 ± 6.6	0.790
	baseline	15.9 ± 3.4	17.3 ± 4.1	
Proteins, %	3-year	17.3 ± 2.1	17.4 ± 2.7	
	5-year	16.7 ± 2.1	16.6 ± 2.1	0.425
	baseline	39.9 ± 7.5	41.4 ± 7.4	
Lipids, %	3-year	39.3 ± 7.5	41.0 ± 7.3	
	5-year	43.1 ± 6.9	42.7 ± 6.0	0.800

Results are expressed as mean ± SD. Two-way analysis of covariance (ANCOVA) was performed after adjustments for age and sex. Data points are significant, *p* < 0.05. * Difference in means between participants in time with respect to baseline. ^#^ Difference in means between participants in time with respect to 3 years. ^$^ Difference in means between groups (reduce or increase their physical activity) at the same time.

**Table 2:** Oxidative stress biomarkers of participants with MetS at baseline, as well as after 3-year and 5-year intervention, according to changes in physical activity.

		Reduce Their Physical Activity (n = 20)	Increase Their Physical Activity (n = 20)	*p*-Value
PBMCs biomarkers				
ROS production stimulated with LPS (RLU/min·10^3^ cells)	baseline	1159.6 ± 567.9	1275.4 ± 814.9	
	3-year	786.2 ± 790.4	961.8 ± 769.8	
	5-year	423.8 ± 308.1 *	351.6 ± 144.7 *^,$^	<0.001
Plasma biomarkers				
	baseline	47.7 ± 17.9	58.2 ± 50.0	
CAT activity (k/L plasma)	3-year	28.7 ± 3.8 *	28.8 ± 6.6 *	
	5-year	29.1 ± 5.3 *	33.6 ± 6.0 *^,#^	<0.001
SOD activity (pkat/L plasma)	baseline	70.7 ± 69.1	112.6 ± 98.5	
	3-year	268.4 ± 60.9 *	263.5 ± 49.0 *	
	5-year	271.8 ± 49.5 *	311.5 ± 49.9 *^,#^	<0.001
	baseline	1.1 ± 1.4	2.1 ± 2.9	
MDA (nM)	3-year	0.6 ± 0.2	0.6 ± 0.2 *	
	5-year	0.6 ± 0.1	0.4 ± 0.1 *^,#^	0.001

Results are expressed as mean ± SD. Two-way analysis of covariance (ANCOVA) was performed after adjustments for age and sex. Data points are significant, *p* < 0.05. * Difference in means between participants in time with respect to baseline. ^#^ Difference in means between participants in time with respect to 3 years. ^$^ Difference in means between groups (reduce or increase their physical activity) at the same time. Abbreviations: PBMCs, peripheral blood mononuclear cells; ROS, reactive oxygen species; LPS, lipopolysaccharide; CAT, catalase; SOD, superoxide dismutase; MDA, malondialdehyde; RLU, relative light units; and MetS, metabolic syndrome.

## Data Availability

The data presented in this study are available on request from the corresponding author due to the signed consent agreements around data sharing. Researchers wishing to access the trial data used in this study can request access from the corresponding author: pep.tur@uib.es.

## References

[1] Saklayen M.G. (2018). The Global Epidemic of the Metabolic Syndrome. Curr. Hypertens. Rep..

[2] O’Neill S., O’Driscoll L. (2015). Metabolic syndrome: A closer look at the growing epidemic and its associated pathologies. Obes. Rev..

[3] Virani S.S., Alonso A., Benjamin E.J., Bittencourt M.S., Callaway C.W., Carson A.P., Chamberlain A.M., Chang A.R., Cheng S., Delling F.N. (2020). Heart Disease and Stroke Statistics—2020 Update: A Report From the American Heart Association. Circulation.

[4] Bellia A., Giardina E., Lauro D., Tesauro M., Di Fede G., Cusumano G., Federici M., Rini G.B., Novelli G., Lauro R. (2009). “The Linosa Study”: Epidemiological and heritability data of the metabolic syndrome in a Caucasian genetic isolate. Nutr. Metab. Cardiovasc. Dis..

[5] Gupta A., Gupta V. (2010). Metabolic syndrome: What are the risks for humans?. Biosci. Trends.

[6] Wu J., Zhang H., Yang L., Shao J., Chen D., Cui N., Tang L., Fu Y., Xue E., Lai C. (2022). Sedentary time and the risk of metabolic syndrome: A systematic review and dose–response meta-analysis. Obes. Rev..

[7] Ang J.-H.C., Sun L., Foo S.-Y.R., Leow M.K.-S., Vidal-Puig A., Fontana L., Dalakoti M. (2025). Perspectives on whole body and tissue-specific metabolic flexibility and implications in cardiometabolic diseases. Cell Rep. Med..

[8] Lemos G.d.O., Torrinhas R.S., Waitzberg D.L. (2023). Nutrients, Physical Activity, and Mitochondrial Dysfunction in the Setting of Metabolic Syndrome. Nutrients.

[9] Onu A., Trofin D.-M., Tutu A., Onu I., Galaction A.-I., Sardaru D.-P., Trofin D., Onita C.A., Iordan D.-A., Matei D.-V. (2025). Integrative Strategies for Preventing and Managing Metabolic Syndrome: The Impact of Exercise and Diet on Oxidative Stress Reduction—A Review. Life.

[10] Birben E., Sahiner U.M., Sackesen C., Erzurum S., Kalayci O. (2012). Oxidative Stress and Antioxidant Defense. World Allergy Organ. J..

[11] Monserrat-Mesquida M., Quetglas-Llabrés M., Capó X., Bouzas C., Mateos D., Pons A., Tur J.A., Sureda A. (2020). Metabolic Syndrome Is Associated with Oxidative Stress and Proinflammatory State. Antioxidants.

[12] Fahed G., Aoun L., Zerdan M.B., Allam S., Zerdan M.B., Bouferraa Y., Assi H.I. (2022). Metabolic Syndrome: Updates on Pathophysiology and Management in 2021. Int. J. Mol. Sci..

[13] Wu J., Fu Y., Chen D., Zhang H., Xue E., Shao J., Tang L., Zhao B., Lai C., Ye Z. (2023). Sedentary behavior patterns and the risk of non-communicable diseases and all-cause mortality: A systematic review and meta-analysis. Int. J. Nurs. Stud..

[14] Myers J., Kokkinos P., Nyelin E. (2019). Physical Activity, Cardiorespiratory Fitness, and the Metabolic Syndrome. Nutrients.

[15] Allard-Chamard H., Mishra H.K., Nandi M., Mayhue M., Menendez A., Ilangumaran S., Ramanathan S. (2020). Interleukin-15 in autoimmunity. Cytokine.

[16] Leal L.G., Lopes M.A., Batista M.L. (2018). Physical exercise-induced myokines and muscle-adipose tissue crosstalk: A review of current knowledge and the implications for health and metabolic diseases. Front. Physiol..

[17] Ainsworth B.E., Haskell W.L., Leon A.S., Jacobs D.R., Montoye H.J., Sallis J.F., Paffenbarger R.S. (1993). Compendium of physical activities: Classification of energy costs of human physical activities. Med. Sci. Sports Exerc..

[18] Fernández-Ballart J.D., Piñol J.L., Zazpe I., Corella D., Carrasco P., Toledo E., Perez-Bauer M., Martínez-González M.Á., Salas-Salvadó J., Martn-Moreno J.M. (2010). Relative validity of a semi-quantitative food-frequency questionnaire in an elderly Mediterranean population of Spain. Br. J. Nutr..

[19] Moreiras O., Carbajal A., Cabrera L., Cuadrado C. (2018). Tablas de Composicion de Alimentos (Ciencia y Tecnica). Guía de Prácticas.

[20] Bouzas C., Bibiloni M.D.M., Julibert A., Ruiz-Canela M., Salas-Salvadó J., Corella D., Zomeño M.D., Romaguera D., Vioque J., Alonso-Gómez Á.M. (2020). Adherence to the Mediterranean Lifestyle and Desired Body Weight Loss in a Mediterranean Adult Population with Overweight: A PREDIMED-Plus Study. Nutrients.

[21] Aebi H. (1984). Catalase in Vitro. Methods Enzym. Anal..

[22] McCord J.M., Fridovich I. (1969). Superoxide dismutase. An enzymic function for erythrocuprein (hemocuprein). J. Biol. Chem..

[23] Capeillère-Blandin C. (1998). Oxidation of guaiacol by myeloperoxidase: A two-electron-oxidized guaiacol transient species as a mediator of NADPH oxidation. Biochem. J..

[24] Ekelund U., Tarp J., Steene-Johannessen J., Hansen B.H., Jefferis B., Fagerland M.W., Whincup P., Diaz K.M., Hooker S.P., Chernofsky A. (2019). Dose-response associations between accelerometry measured physical activity and sedentary time and all cause mortality: Systematic review and harmonised meta-analysis. BMJ.

[25] Matta L., de Faria C.C., De Oliveira D.F., Andrade I.S., Lima-Junior N.C., Gregório B.M., Takiya C.M., Ferreira A.C.F., Nascimento J.H.M., de Carvalho D.P. (2022). Exercise Improves Redox Homeostasis and Mitochondrial Function in White Adipose Tissue. Antioxidants.

[26] Clemente-Suárez V.J., Rubio-Zarapuz A., Belinchón-deMiguel P., Beltrán-Velasco A.I., Martín-Rodríguez A., Tornero-Aguilera J.F. (2024). Impact of Physical Activity on Cellular Metabolism Across Both Neurodegenerative and General Neurological Conditions: A Narrative Review. Cells.

[27] Kolodziej F., O’Halloran K.D. (2021). Re-Evaluating the Oxidative Phenotype: Can Endurance Exercise Save the Western World?. Antioxidants.

[28] Pesta D., Roden M. (2017). The Janus Head of Oxidative Stress in Metabolic Diseases and During Physical Exercise. Curr. Diab. Rep..

[29] Gómez-Cabrera M.C., Salvador-Pascual A., Cabo H., Ferrando B., Viña J. (2015). Redox modulation of mitochondriogenesis in exercise. Does antioxidant supplementation blunt the benefits of exercise training?. Free Radic. Biol. Med..

[30] Powers S.K., Deminice R., Ozdemir M., Yoshihara T., Bomkamp M.P., Hyatt H. (2020). Exercise-induced oxidative stress: Friend or foe?. J. Sport Health Sci..

[31] El Assar M., Álvarez-Bustos A., Sosa P., Angulo J., Rodríguez-Mañas L. (2022). Effect of Physical Activity/Exercise on Oxidative Stress and Inflammation in Muscle and Vascular Aging. Int. J. Mol. Sci..

[32] Scarfò G., Daniele S., Chelucci E., Rizza A., Fusi J., Freggia G., Costa B., Taliani S., Artini P., Martini C. (2023). Regular exercise delays microvascular endothelial dysfunction by regulating antioxidant capacity and cellular metabolism. Sci. Rep..

[33] Nani A., Murtaza B., Sayed Khan A., Khan N.A., Hichami A. (2021). Antioxidant and Anti-Inflammatory Potential of Polyphenols Contained in Mediterranean Diet in Obesity: Molecular Mechanisms. Molecules.

[34] Chen S.-J.J., Yen C.-H.H., Huang Y.-C.C., Lee B.-J.J., Hsia S., Lin P.-T.T. (2012). Relationships between Inflammation, Adiponectin, and Oxidative Stress in Metabolic Syndrome. PLoS ONE.

[35] Quetglas-Llabrés M.M., Monserrat-Mesquida M., Bouzas C., García S., Mateos D., Ugarriza L., Gómez C., Sureda A., Tur J.A. (2024). Long-Term Impact of Nutritional Intervention with Increased Polyphenol Intake and Physical Activity Promotion on Oxidative and Inflammatory Profiles in Patients with Metabolic Syndrome. Nutrients.

[36] Moreto F., Kano H.T., Torezan G.A., de Oliveira E.P., Manda R.M., Teixeira O., Michelin E., Correa C.R., Burini R.C. (2015). Changes in malondialdehyde and C-reactive protein concentrations after lifestyle modification are related to different metabolic syndrome-associated pathophysiological processes. Diabetes Metab. Syndr. Clin. Res. Rev..

[37] Rytz C.L., Pialoux V., Mura M., Martin A., Hogan D.B., Hill M.D., Poulin M.J. (2020). Impact of aerobic exercise, sex, and metabolic syndrome on markers of oxidative stress: Results from the Brain in Motion study. J. Appl. Physiol..

[38] Klebanoff S.J. (2005). Myeloperoxidase: Friend and foe. J. Leukoc. Biol..

[39] Tumova E., Sun W., Jones P.H., Vrablik M., Ballantyne C.M., Hoogeveen R.C. (2013). The Impact of Rapid Weight Loss on Oxidative Stress Markers and the Expression of the Metabolic Syndrome in Obese Individuals. J. Obes..

[40] Mathew A.V., Li L., Byun J., Guo Y., Michailidis G., Jaiswal M., Chen Y.E., Pop-Busui R., Pennathur S. (2018). Therapeutic Lifestyle Changes Improve HDL Function by Inhibiting Myeloperoxidase-Mediated Oxidation in Patients With Metabolic Syndrome. Diabetes Care.

[41] Li F., Li Y., Tang Y., Lin B., Kong X., Oladele O.A., Yin Y. (2014). Protective effect of myokine IL-15 against H_2_O_2_-mediated oxidative stress in skeletal muscle cells. Mol. Biol. Rep..

[42] Gonzalez-Gil A.M., Elizondo-Montemayor L. (2020). The Role of Exercise in the Interplay between Myokines, Hepatokines, Osteokines, Adipokines, and Modulation of Inflammation for Energy Substrate Redistribution and Fat Mass Loss: A Review. Nutrients.

[43] Pérez-López A., Valadés D., Vázquez Martínez C., de Cos Blanco A.I., Bujan J., García-Honduvilla N. (2018). Serum IL-15 and IL-15Rα levels are decreased in lean and obese physically active humans. Scand. J. Med. Sci. Sports.

[44] Cho E., Chodzko M., Compton S.L.E., Yang S., Heymsfield S., Spielmann G., Brown J.C. (2025). Effects of aerobic exercise on body composition and exerkines in colorectal cancer survivors. Front. Sport. Act. Living.

[45] Luo Z., He Z., Qin H., Chen Y., Qi B., Lin J., Sun Y., Sun J., Su X., Long Z. (2022). Exercise-induced IL-15 acted as a positive prognostic implication and tumor-suppressed role in pan-cancer. Front. Pharmacol..

[46] Della Guardia L., Codella R. (2021). Exercise tolls the bell for key mediators of low-grade inflammation in dysmetabolic conditions. Cytokine Growth Factor Rev..

[47] Bellicha A., van Baak M.A., Battista F., Beaulieu K., Blundell J.E., Busetto L., Carraça E.V., Dicker D., Encantado J., Ermolao A. (2021). Effect of exercise training on weight loss, body composition changes, and weight maintenance in adults with overweight or obesity: An overview of 12 systematic reviews and 149 studies. Obes. Rev..

[48] Richter E.A. (2021). Is GLUT4 translocation the answer to exercise-stimulated muscle glucose uptake?. Am. J. Physiol. Metab..

[49] Ostman C., Jewiss D., Smart N.A. (2017). The Effect of Exercise Training Intensity on Quality of Life in Heart Failure Patients: A Systematic Review and Meta-Analysis. Cardiology.

[50] Okura T., Nakata Y., Ohkawara K., Numao S., Katayama Y., Matsuo T., Tanaka K. (2007). Effects of Aerobic Exercise on Metabolic Syndrome Improvement in Response to Weight Reduction. Obesity.

[51] Share B.L., Naughton G.A., Obert P., Peat J.K., Aumand E.A., Kemp J.G. (2015). Effects of a Multi-Disciplinary Lifestyle Intervention on Cardiometabolic Risk Factors in Young Women with Abdominal Obesity: A Randomised Controlled Trial. PLoS ONE.

[52] Gondim O.S., de Camargo V.T.N., Gutierrez F.A., Martins P.F.d.O., Passos M.E.P., Momesso C.M., Santos V.C., Gorjão R., Pithon-Curi T.C., Cury-Boaventura M.F. (2015). Benefits of Regular Exercise on Inflammatory and Cardiovascular Risk Markers in Normal Weight, Overweight and Obese Adults. PLoS ONE.

[53] Brandt C., Pedersen B.K. (2022). Physical Activity, Obesity and Weight Loss Maintenance. From Obesity to Diabetes.

[54] Liu Y., Wang X., Fang Z. (2024). Evaluating the impact of exercise on intermediate disease markers in overweight and obese individuals through a network meta-analysis of randomized controlled trials. Sci. Rep..

[55] Xing S., Zhang Y., Chen Y., Feng S., Zhang Y., Moreira P. (2025). Comparing the impacts of different exercise interventions on patients with type 2 diabetes mellitus: A literature review and meta-analysis. Front. Endocrinol..

[56] Franczyk B., Gluba-Brzózka A., Ciałkowska-Rysz A., Ławiński J., Rysz J. (2023). The Impact of Aerobic Exercise on HDL Quantity and Quality: A Narrative Review. Int. J. Mol. Sci..

[57] Sayón-Orea C., Razquin C., Bulló M., Corella D., Fitó M., Romaguera D., Vioque J., Alonso-Gómez Á.M., Wärnberg J., Martínez J.A. (2019). Effect of a Nutritional and Behavioral Intervention on Energy-Reduced Mediterranean Diet Adherence Among Patients With Metabolic Syndrome. JAMA.

[58] Martucci M., Ostan R., Biondi F., Bellavista E., Fabbri C., Bertarelli C., Salvioli S., Capri M., Franceschi C., Santoro A. (2017). Mediterranean diet and inflammaging within the hormesis paradigm. Nutr. Rev..

